# Case Report: Immediate preprocedural CT as a decision-making checkpoint before middle meningeal artery embolization for traumatic epidural hematoma

**DOI:** 10.3389/fsurg.2026.1840411

**Published:** 2026-05-19

**Authors:** Guangliang Zhang, Jing Zhang, Ruyong Ye, Kuang Zheng, Ming Zhong

**Affiliations:** 1 Department of Neurosurgery, Yongkang First People’s Hospital of Wenzhou Medical University, Yongkang, Zhejiang, China; 2 Department of Neurosurgery, The First Affiliated Hospital of Wenzhou Medical University, Wenzhou, Zhejiang, China

**Keywords:** endovascular treatment, intracranial hemorrhage, middle meningeal artery embolization, preprocedural CT, traumatic epidural hematoma

## Abstract

**Background:**

Traumatic epidural hematoma (EDH) is typically managed surgically when associated with neurological deterioration or significant mass effect. In neurologically intact patients, conservative management or minimally invasive approaches such as middle meningeal artery (MMA) embolization may be considered. The role of periprocedural imaging in guiding endovascular decision-making remains to be clarified.

**Case presentation:**

A neurologically intact patient with a left-sided traumatic EDH underwent planned MMA embolization. Immediate preprocedural noncontrast computed tomography (CT) was not performed prior to angiography. Embolization of the left MMA was successfully completed. Postprocedural CT revealed a newly developed contralateral parietal EDH. Repeat angiography confirmed dural arterial supply from the right MMA, and contralateral embolization was performed during the same session. The patient remained clinically stable and did not require surgical evacuation. Follow-up imaging demonstrated stabilization and gradual resolution of both hematomas.

**Conclusion:**

We hypothesize that immediate preprocedural CT may aid decision-making for MMA embolization in selected EDH cases, though this single observation requires validation and is not practice-guiding.

## Introduction

Traumatic epidural hematoma (EDH) is defined as the accumulation of blood between the inner table of the skull and the dura mater, typically resulting from middle meningeal artery (MMA) injury. While surgical evacuation remains the cornerstone of management for lesions associated with neurological deterioration or significant mass effect (1), the optimal strategy for neurologically intact patients whose hematomas do not meet operative thresholds remains an active area of investigation.

Recently, MMA embolization has emerged as a minimally invasive adjunct or alternative for selected traumatic extra-axial hemorrhage, informed by broader experience in chronic subdural hematoma management (2, 3). However, its specific role in acute traumatic EDH remains investigational, with evidence largely limited to small series and case-based reports (4, 5).

Standardized preprocedural workflows remain ill-defined, particularly regarding imaging checkpoints used to guide endovascular decision-making (6). Although delayed or contralateral EDH is a recognized phenomenon typically detected on interval imaging (7), the utility of imaging reassessment immediately before endovascular intervention remains less well defined. This study explores the potential value of immediate preprocedural noncontrast CT as a decision-making checkpoint.

## Case presentation

A 57-year-old male with no significant medical history and no regular medication use was admitted approximately 30 min after being struck by a motor vehicle as a pedestrian. He presented with transient loss of consciousness, post-traumatic amnesia, nausea, vomiting, and severe diffuse pain.

On initial examination, vital signs revealed stress-induced hypertension (172/121 mmHg) and tachycardia (109 bpm). Neurological assessment showed a Glasgow Coma Scale (GCS) score of 15 (E4V5M6). Cranial nerves were intact, with bilaterally equal and reactive pupils. Motor strength was grossly intact; however, formal testing was precluded by pain from polytrauma.

**Figure 1:**
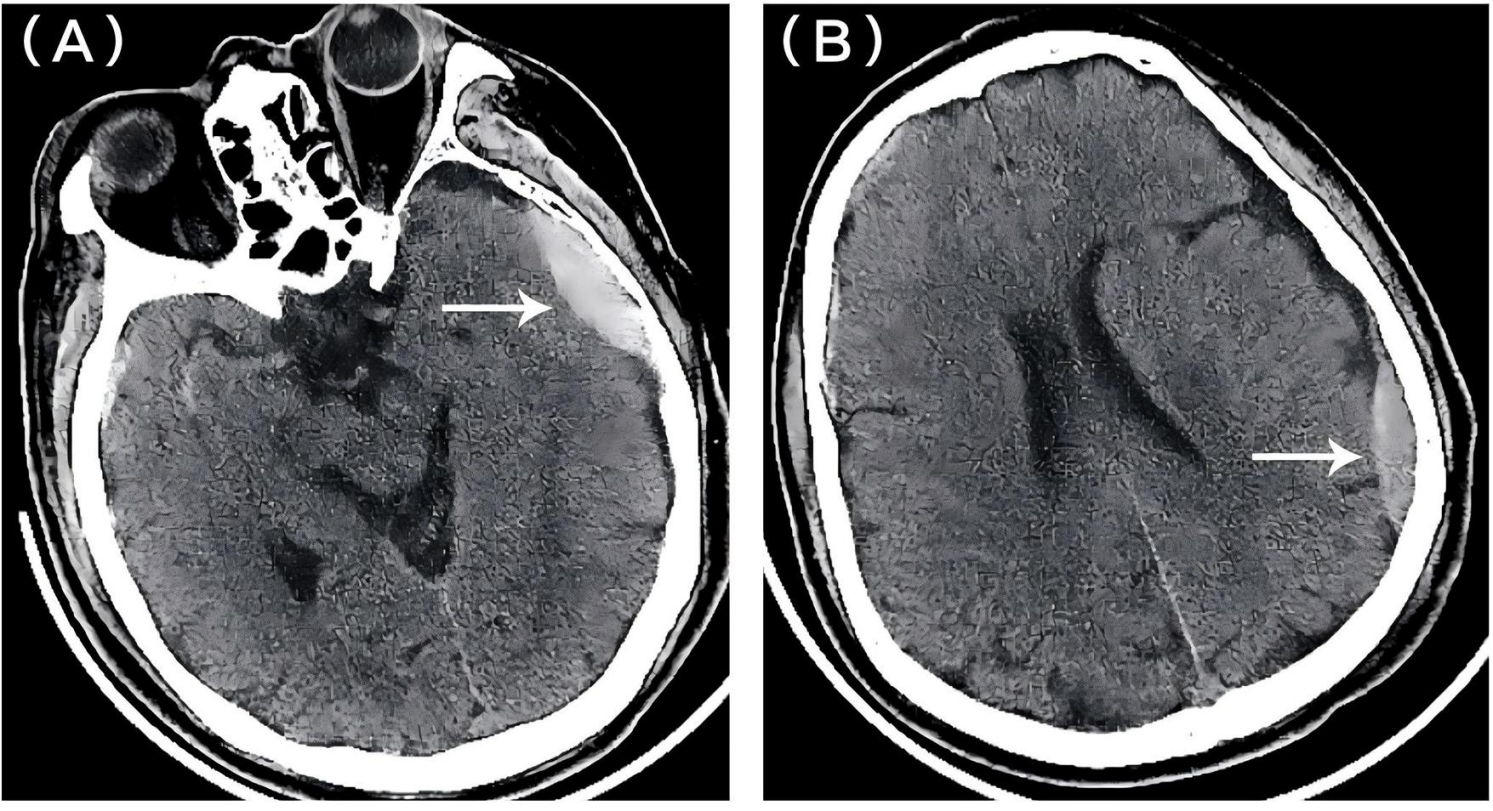
Initial noncontrast head CT demonstrating a left-sided epidural hematoma (EDH). **(A)** Axial CT image at the basal skull level (lower slice) showing the epidural hematoma (arrow) with a maximal thickness of approximately 1.2 cm. **(B)** Axial CT image at the level of the lateral ventricles (higher slice) revealing the epidural hematoma (arrow), which appears thinner. There is no significant mass effect or midline shift in either slice.

Neuroimaging demonstrated a left temporo-occipital epidural hematoma (EDH) without significant mass effect (Figures 1A,B), alongside a small right frontotemporal subdural hematoma and scattered subarachnoid hemorrhage. Further diagnostic imaging confirmed multiple traumatic injuries, including a left scapular fracture, non-displaced left rib fractures (4th–7th), L1–L4 transverse process fractures, and mild left lower lobe pulmonary contusion.

### Management strategy

Although the initial EDH did not meet the absolute threshold for craniotomy, recent studies have suggested that conservative management of hyperacute EDH may carry a substantial risk of hematoma expansion (up to 42.3%) and a considerable conversion rate to craniotomy (up to 23%) (5). However, these estimates are derived from a relatively small cohort, and thus should be interpreted with caution. Middle meningeal artery (MMA) embolization was therefore pursued as a preemptive protective measure. In this particular case, stress-induced hypertension and persistent vomiting further increased the risk of progression, while his polytrauma status raised the anesthetic risks of open surgery.

### Procedure and follow-up

To clarify the chronology of events and address the potential etiology of the contralateral hematoma, the precise timeline was defined as follows:

1. Injury to Initial CT:∼30 min
2. Initial CT to Arrival at Angiography Suite:∼30 min (60 min post-injury).
3. Initial CT to Detection of Contralateral EDH:∼90 min (2 h post-injury).

**Figure 2:**
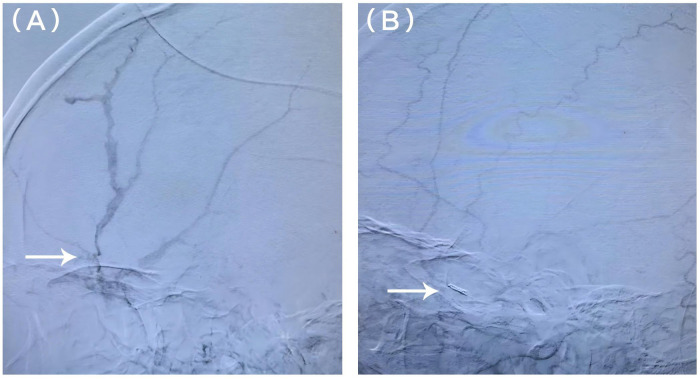
Angiographic demonstration of interventional embolization for epidural hematoma. **(A)** Digital subtraction angiographic image (arterial phase) showing the middle meningeal artery (arrow) supplying the epidural hematoma. **(B)** Post-embolization angiographic image showing successful occlusion of the middle meningeal artery by an embolic agent (arrow).

**Figure 3:**
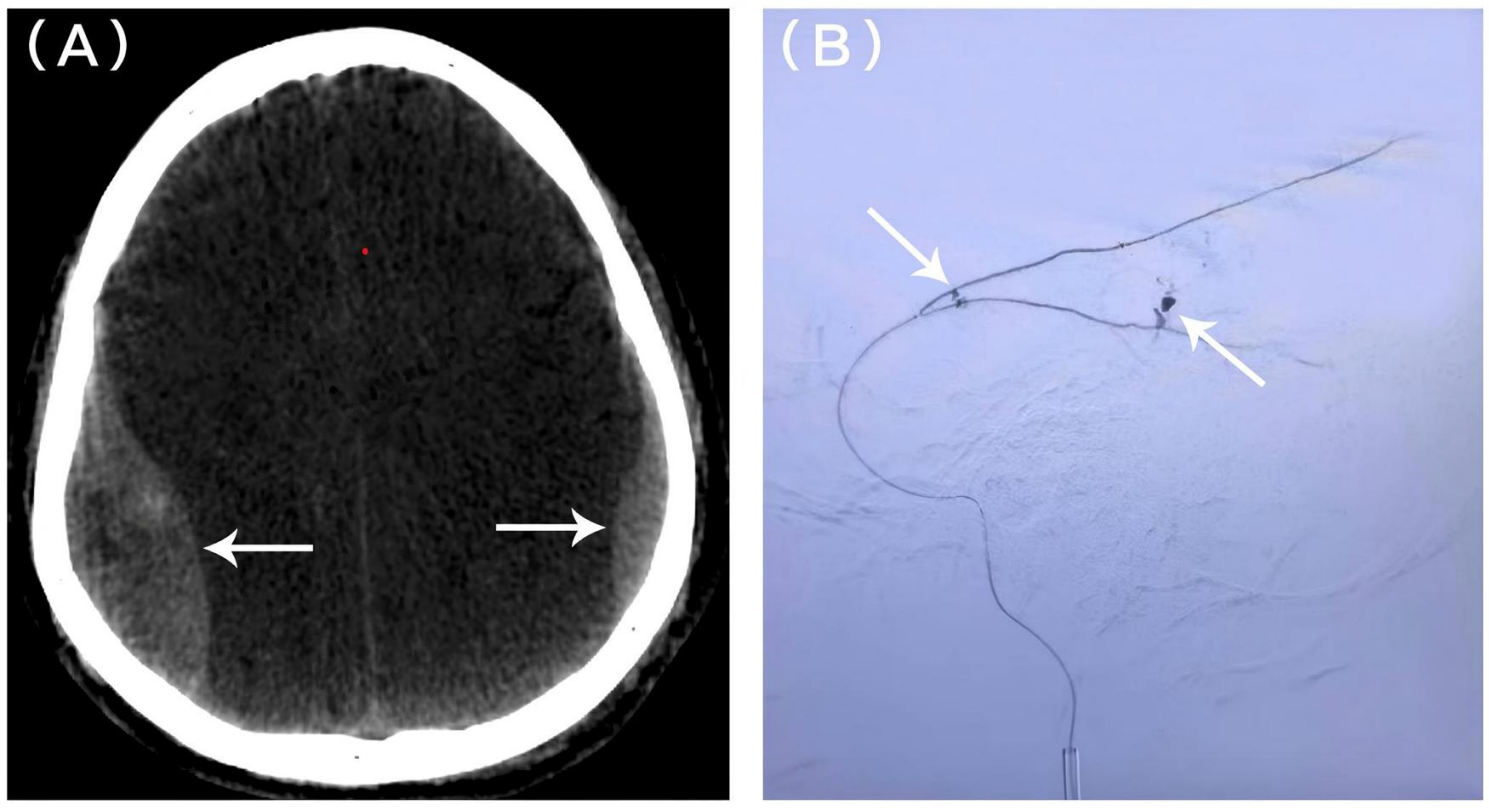
Interval development of contralateral EDH detected after the initial embolization. **(A)** Axial noncontrast CT image obtained after the initial embolization showing a newly developed epidural hematoma in the right parietal region (arrow). **(B)** Digital subtraction angiographic image of the right middle meningeal artery (MMA) demonstrating dural arterial supply to the contralateral (right) epidural hematoma (arrow).

The patient was transferred to the DSA suite shortly after initial imaging. Left MMA embolization was successfully performed 2 h post-injury (Figures 2A,B). Immediate post-embolization CT unexpectedly revealed a newly developed right parietal EDH (Figure 3A).Given the rapid interval change, right MMA angiography was performed, demonstrating dural arterial supply (Figure 3B),Consequently, right MMA embolization was performed emergently during the same session 2.5 h post-injury. The patient remained neurologically intact throughout the hospital course. Due to the need for definitive orthopedic management, he was transferred to another facility. At the 3-month telephone follow-up, the patient reported a good recovery with complete resolution of headache and no new neurological deficits. External hospital follow-up CT was reviewed, confirming complete resolution of the intracranial hematomas (GOS 5).

## Discussion

This case does not introduce a novel pathological entity nor redefine treatment indications for traumatic EDH. Instead, it highlights imaging timing within the endovascular workflow, identifying immediate preprocedural noncontrast CT as a potentially underrecognized decision-making checkpoint that warrants further investigation.

Delayed and contralateral EDH are well documented after head trauma and may occur even in neurologically stable patients (7). In the present case, the absence of immediate preprocedural CT prior to angiography delayed the recognition of a contralateral hematoma. While this reflected routine local practice, it underscores the variability in institutional workflows regarding repeat imaging (6).

### Critical appraisal of etiology

While the precise cause of the contralateral EDH remains undetermined, a critical appraisal is necessary. First, the possibility of a missed lesion on the initial CT cannot be excluded: a very small hematoma might have been present but below the threshold of detection. Second, although systemic heparinization was not utilized, physiological factors such as stress-induced hypertension (admission 172/121 mmHg, maintained at 140 to 170 mmHg intraoperatively) and hemodynamic fluctuations during embolization could theoretically have promoted hemorrhage. Third, while routine coagulation profiles (PT/INR, PTT, platelets) were within normal limits and there was no clinical evidence of overt coagulopathy, comprehensive platelet function testing was not performed.

The presence of polytrauma may theoretically predispose to consumptive coagulopathy or inflammatory cascades affecting hemostasis. Therefore, the development of the contralateral EDH is likely multifactorial.

### Clinical considerations for the proposed workflow adjustment

Despite the favorable outcome, detecting the hematoma would likely have significantly optimized the interventional strategy.

Prioritization of Embolization Targets: If the right-sided EDH had been identified and demonstrated faster progression or a larger volume, the primary intervention target would logically have shifted. Embolization of the right MMA would have taken priority to stabilize the more aggressive lesion first, followed by addressing the left side.

Dynamic Reassessment of Surgical Indications: Early identification of rapidly expanding hematomas would have triggered an immediate multidisciplinary reassessment. Had any hematoma surpassed the surgical thresholds, upon discovery, the strategy would have shifted directly to craniotomy rather than endovascular therapy.

Improvement of Procedural Efficiency: Recognizing both lesions initially would have allowed for a single, comprehensive angiography session. This would eliminate the need for a repeat emergent procedure, thereby reducing overall procedural time, radiation and contrast agent exposure, which represents a key opportunity for workflow optimization highlighted in recent multinational surveys (6).

Therefore, the value of this protocol lies in facilitating a nuanced and prioritized treatment stratification, based as it is on a single case. Consequently, no causal inference can be drawn, and it should be interpreted cautiously as a hypothesis-generating finding pending future validation.

## Conclusion

**Figure 4:**
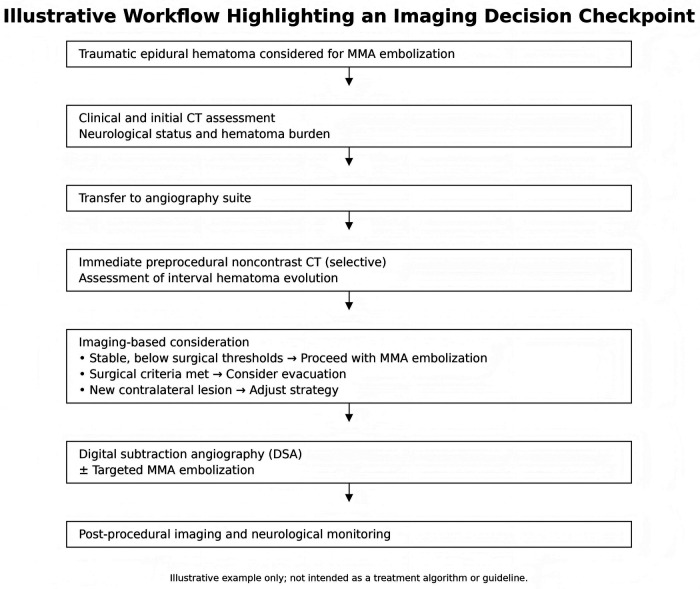
Exploratory workflow illustrating immediate preprocedural CT as a decision-making checkpoint prior to MMA embolization.

This single-case observation suggests that selective immediate preprocedural noncontrast CT could represent a potential decision-making checkpoint in patients with traumatic EDH undergoing MMA embolization (Figure 4). However, given the inherent limitations of a case report, no causal inferences regarding its impact on patient outcomes can be drawn, and these findings should not be interpreted as prescriptive clinical guidance. Future validation through larger, prospective studies is required.

## Data Availability

The original contributions presented in the study are included in the article/Supplementary Material, further inquiries can be directed to the corresponding authors.
